# Women’s lived experiences of the built environment and maternal psychosocial well-being in urban slums and slum rehabilitation colonies: a qualitative study in Bhopal, India

**DOI:** 10.1080/16549716.2026.2721061

**Published:** 2026-09-02

**Authors:** Kamini Charan, Vikas Yadav, Krushna Chandra Sahoo, Uday K. Mandal, Yogesh D. Sabde, Rajnarayan R. Tiwari, Vishal Diwan

**Affiliations:** a Division of Environmental Monitoring and Exposure Assessment (Water and Soil), ICMR National Institute for Research in Environmental Health (ICMR-NIREH), Bhopal, India; b Department of Health Sciences, Savitribai Phule Pune University, Pune, India; c Division of Environmental Health and Epidemiology, ICMR National Institute for Research in Environmental Health (ICMR-NIREH), Bhopal, India; d Faculty of Medical Sciences, Academy of Scientific & Innovative Research (AcSIR), Ghaziabad, India; e Health Technology Assessment India, Department of Health Research, Ministry of Health & Family Welfare, Government of India, New Delhi, India; f ICMR National Institute for Research in Environmental Health (ICMR-NIREH), Bhopal, India; g Department of Global Public Health, Karolinska Institute, Stockholm, Sweden

**Keywords:** Housing quality, neighbourhood environment, urban poverty, women’s health, phenomenology

## Abstract

**Background:**

Nearly half of India’s urban population lives in informal settlements, facing substandard living conditions due to overcrowding, poor infrastructure, and limited access to basic amenities. These conditions critically affect the psychosocial well-being of mothers, yet limited research exists on how the built environment impacts maternal psychosocial health in urban poor settings in India.

**Objective:**

To explore the influence of housing conditions and neighborhood environments on maternal psychosocial health among mothers of children under five residing in urban slums and in-situ slum rehabilitation colonies in Bhopal, India.

**Methods:**

A qualitative study using a phenomenological approach was conducted through focus group discussions and in-depth interviews with mothers from both slum and in-situ slum rehabilitation settings. Thematic analysis was employed to identify emerging patterns and interpretive themes reflecting shared and lived experiences.

**Results:**

Women residing in slum areas were particularly vulnerable due to overcrowded and poorly constructed dwellings. Inadequate infrastructure and environmental stressors contributed to persistent fears of accidents and safety risks. In both slum and BSUP rehabilitation contexts, social isolation and limited community cohesion heightened fears of crime, adversely affecting psychosocial health. Uncertainty surrounding potential relocation, combined with caregiving-related health concerns, further intensified emotional stress and psychosocial burden.

**Conclusions:**

The findings highlight the need to improve housing quality, ensure reliable access to essential amenities, and strengthen community support systems. Developing comprehensive, context-sensitive strategies that enhance resilience and psychosocial well-being is essential for promoting inclusive and sustainable urban development among vulnerable populations.

## Paper Context

**Main findings**: Poor housing conditions, neighbourhood infrastructure, environmental hazards, and weak social cohesion contribute to maternal psychosocial distress among women living in urban slums and slum rehabilitation colonies in Bhopal, India.

**Added knowledge**: This qualitative study demonstrates that maternal psychosocial well-being is influenced not only by housing quality but also by neighbourhood environments and social relationships, highlighting that relocation to improved housing alone may not reduce psychosocial distress.

**Global health impact for policy and action**: The findings support integrated urban policies that improve housing quality, neighbourhood infrastructure, sanitation, environmental conditions, and community support to promote maternal psychosocial well-being among vulnerable urban populations.

## Background

Internal migration in India, where an estimated 42% of the population travels within the country, is fuelled by urbanisation and economic differences among states. Nearly 2.4 million individuals migrate to Madhya Pradesh state each year for employment [1]. This mass migration brings availability and affordability challenges to critically constrained resources such as housing, water supply, and healthcare [2]. This has often led to the emergence of low-cost informal settlements (slums). According to World Bank estimates, approximately 49% of India’s urban population lives in slum-like conditions [3]. To curb these challenges, the government of India introduced the Basic Services for Urban Poor (BSUP) – slum rehabilitation colony under the Jawaharlal Nehru National Urban Renewal Mission in 2005 [4–6]. Aiming to provide secure and stable housing with access to basic amenities such as water, sanitation, and electricity [6,7]. However, case studies across 11 major BSUP projects reveal houses and amenities provided are unsatisfactory and that rehabilitated families feel displaced [8–11].

Globally, rapid urbanisation and housing insecurity such as overcrowding, inadequate sanitation, and exposure to environmental hazards contribute to poor living conditions [12,13]. These factors have been linked to issues like stress, anxiety, and emotional distress among mothers, impacting their overall well-being and caregiving abilities [12]. Environmental stressors in slums, including exposure to noise pollution, heat, lack of privacy, and fear of eviction, contribute to maternal distress [14] and their ability to care for children effectively, leading to a vicious cycle of poverty and poor health outcomes [15–21]. Focusing on women residing in urban slums, a study by Jungari et al. states that lower levels of education among migratory women lead to a lack of participation in the formal employment sector, along with gender discrimination [22]. Whereas mothers carry a dual burden of child rearing and managing household responsibilities, often without adequate support, which in turn creates a barrier to education and affects overall health [23–27], which emphasised the need for in-depth understanding.

This study is based on a socio-ecological view of health, which acknowledges that maternal psychosocial well-being is influenced by the interaction of physical housing conditions, neighbourhood infrastructure, and social contexts. In this study, psychosocial health is characterised as a comprehensive concept encompassing emotional distress (including stress, fear, and worry), perceived safety, social connectedness, and the capacity to manage daily demands within one’s living environment. The BSUP is a planned settlement that includes improved basic dwellings and expected access to key services. This programmatic intervention aims to reduce environmental stressors, allowing for more accurate attribution of psychosocial consequences connected to the built environment. However, informal, unplanned communities with high densities are more vulnerable to environmental pressures. As a result, comparing the two contexts reveals how planned housing in rehabilitation colonies reduces maternal psychosocial risk factors as compared to informal slums, where environmental constraints may impact maternal well-being. Therefore, we investigated and sought to compare the perspectives and lived experiences of mothers of children under the age of five living in a rehabilitation colony built by BSUP and a conventional slum in Bhopal, India. The study aims to develop context-specific insights for improved urban health and housing policy by examining how distinct architectural and social contexts influence mothers’ psychosocial well-being.

## Methods

### Study design and setting

We conducted a qualitative study using a phenomenological approach. This study intended to understand the essence of phenomena in the case of the built environment – housing and availability of basic amenities among the urban poor and maternal psychosocial risk factors – rather than measure external correlations or construct a concept or theory. It explored the lived experiences of new mothers’ perceptions and how they make meaning of their built environment in relation to their psychosocial health. This approach describes underlying experiences and interpretive themes across participants to reveal shared and divergent essences with a nuanced, in-depth understanding of how urban poor settings shape maternal psychosocial well-being, informing context-sensitive interventions and policies.

The study setting comprised traditional slums and the in-situ slum rehabilitation colonies from the Bhopal Municipal Corporation. It consists of 85 wards with a population of 1.9 million, and around 27% live in informal settlements or slums. The city has a high migration rate and diverse socio-cultural dynamics [28].

### Participant recruitment

A total of 27 women, who have children under 5 years of age and reside in the rehabilitation colony under BSUP and the traditional slum, participated in this study. Among them, 12 were from slum households, and 15 were from the BSUP colony. We used purposive sampling. Participants were pre-identified as a part of the quantitative data collection carried out prior. They were recruited by the research team with the support of community volunteers, including Anganwadi workers and Accredited Social Health Activists. Initially, we approached 41 women; 14 were unwilling to participate because of their busy schedules. Detailed participant characteristics are presented in Table 1.

**Table 1.** Participants’ characteristics.

	Focus group discussions		In-depth interviews	
	BSUP colony	Slum	BSUP colony	Slum
No. of participants	9	6	6	6
Age (years)	23–27	21–36	24–35	22–35
Family size	2–11	4–7	4–12	3–8
Number of children	1–3	1–3	1–3	1–2
Youngest child’s age	5 months − 4 years	4 months − 2 years	1–4 years	5 months − 4 years
Number of rooms and housing types	2 Pucca house	1–2 Kuccha/tin house	2 Pucca house	1–2 Kuccha/tin house
Duration of residence	3–11 years	4–15 years	8 months −10 years	4–14 years

### Data collection methods

Data collection was conducted in two phases. During the initial phase (November 2024 to January 2025), one focus group discussion (FGD) was conducted in a slum, and 12 in-depth interviews (IDIs) were conducted, with six IDIs each from the slum and the BSUP colony. Although a second FGD in the BSUP colony was originally planned, it could not be conducted during the initial data collection because of logistical and participant availability constraints. Following reviewer comments during manuscript revision, the second FGD was conducted in the BSUP colony in April 2026. A total of 6 to 9 women participated in each FGD (*n* = 15). The additional FGD followed the same interview guide and purposive sampling approach as the initial FGD and was undertaken to further explore community-level perspectives and strengthen the qualitative findings without changing the study objectives.

The FGD and IDI guides consisted of open-ended questions with probes related to housing conditions, external environmental features, community social interactions, and the connections between these factors (Supplementary file). Interviews were conducted at community centres or participants’ homes, depending on participants’ preference for privacy and convenience during routine vaccination days. The average duration of both FGDs and IDIs was approximately 40 minutes. Handwritten field notes were taken in addition to audio recording. 

We conducted FGDs to discuss major community-level concerns regarding safety and social support of the built environment. The psychosocial health themes frequently prompted individuals to disclose their personal experiences of stigmatisation within their communities. During the early FGD, participants expressed feelings of anxiety and defensiveness. Therefore, we transitioned to IDIs to obtain comprehensive information and ensure the safety of participants, which provided the opportunity to ask sensitive questions while respecting their privacy. The FGDs addressed community-level issues, such as neighbourhood functioning, people’s attitudes toward the constructed environment, and social norms. However, the study underscored the profundity of individual experiences in relation to sensitive maternal psychosocial health, while also reflecting specific group norms. Therefore, this study further explored a diverse range of in-depth experiences to document a diverse array of sensitive narratives, such as stress, stigma, and coping mechanisms, that would not be exposed in a group setting, which provided methodological triangulation. The IDIs provided additional depth in areas where content overlapped. The sampling process was able to identify themes that were common to all groups by ensuring that there were variations in age, parity, manner of housing, and ward. This process continued until no new dimension to the studied phenomena emerged and data saturation was attained.

### Data management and analysis

We transcribed the audio recordings verbatim and translated them into English, ensuring the original meaning and context remained intact. During the data familiarisation process, thematic analysis was identified as a suitable approach based on the type and content of the collected data [29]. Analysis was conducted using an inductive approach, where codes and themes were derived directly from the data rather than being fitted into a pre-existing theoretical framework. This approach was considered most appropriate as it allowed for exploration of maternal perceptions because psychosocial impacts of slum and BSUP built environments remain under-researched. The analysis was performed using both manifest and latent levels, focusing on the explicit and surface as well as interpretive meanings of the participants’ narratives. The authors’ different educational and work experiences in public health helped them better understand and interpret the findings. Preliminary data analysis was carried out simultaneously with data collection; as no new information emerged, data saturation was declared.

Coding and analysis of the data were carried out in MAXQDA 2018 (Universal for Windows and macOS). All identifying information was removed to ensure participant anonymity. Preliminary codes were derived from the data itself and refined through an iterative process of comparison and analysis. Codes falling under the same context and having a significant relationship were grouped into suitable categories and further into subthemes (Table 2). Finally, we found important themes and patterns by looking at the coded data. These helped us understand the key factors that affect mothers’ psychosocial health in relation to their surroundings. We followed the Consolidated Criteria for Reporting Qualitative Research (COREQ) for the reporting of the study [30] (Supplementary file).

**Table 2.** Coding tree with themes, sub-themes and codes.

Themes	Structural vulnerability and housing safety		Neighbourhood infrastructure and safety			Social cohesion, social isolation and neighbourhood security	
Sub-themes	Fear due to poor housing conditions	Emotional exhaustion and stress from overcrowding	Fear of accidents caused by lack of supporting infrastructure	Stress-related to improper waste disposal and open drainage	Anxiety and stress over health, accessibility and relocation	Lack of communal feeling and social isolation	Fear of crime and neighbourhood insecurity
Codes	Homes made of plastic and metal sheets	Presence of extended family	Steep slopes	Open drains	Low blood Pressure	Do not talk much	Men with drinking problems
	Leakage in walls and ceilings	Small size of home	No Pathways and roads	Disposal of garbage in drains	Anaemia	Not allowed to talk to neighbours	Taking away children
	Over heating	Small business from home	No street lights	Overflow during monsoon	Child not gaining weight	Constant fights	Alcohol sold in colony premises
	Poor material used for building BSUP	Unable to relax	Difficulty faced in accessibility during pregnancy due to swollen feet	Children and adults falling into drains	Unable to sleep	People from different caste and religion	Suicide
	Fear of intrusion	Affected child rearing	Difficulty walking up and down the slope during monsoon	No door-to-door waste collection	Constant dizziness	Unable to mingle	Thefts
	Fear of Structure collapsing	Lack of privacy	Broken stairs	Inaccessible garbage bins	Child recurringly falling ill	Can’t share problems with them	Fights and quarrels
	No timely maintenance	Lack of space	No safety grill	Burning of garbage	Willing to live in slum rather than relocating to BSUP	Remarks about poor condition of home	Use of guns
		Suffocation	Fear of children falling	Garbage thrown in open spaces	Fear of losing house in slum		No faith in law enforcement
			Accessibility issues during emergency	Alternate day water supply	Perceived lack of safety and basic facilities in BSUP		Constant need to stay alert
			No elevators		Poor outlook towards BSUP		

## Results

The majority of participants were young women, with ages ranging from 21 to 36 years, and most clustered in their mid-twenties. Participants in BSUP areas predominantly resided in pucca houses with two rooms, whereas those in slum areas lived in semi-pucca or kuccha structures, typically with one to two rooms. Overall, households in slum settings had fewer rooms, often limited to a single-room dwelling.

Three major themes emerged from the analysis: 1) Structural vulnerability and housing safety, 2) Neighbourhood infrastructure and safety, and 3) Social cohesion, social isolation and neighbourhood security.

### Theme 1. Structural vulnerability and housing safety

#### Sub-theme 1.1. Anxiety and fear due to poor housing conditions

Many women reported experiencing anxiety and distress due to the structural instability of their living place. Participants from the slum most commonly mentioned that homes across the slum are partially built with inadequate materials such as plastic sheets. Some participants reported living in houses made of metal sheets and expressed concerns about roof leaks during the monsoon and overheating during the summer. The loose fitting of plastic or metal sheets on roofs and walls further contributed to fears of roof leaks, excessive heat, and potential structural collapse in slum housing. Moreover, the poor infrastructure in slums and BSUP, including damaged walls, broken doors, and water leakage, contributed to a persistent sense of insecurity and vulnerability. Some participants also expressed that the structure could collapse due to its poor construction and lack of maintenance.

*Particularly, during the rainy days, I am afraid of cracks in the walls. I’m afraid that if there is heavy wind, the roof will fall on my children and me. The fear never leaves; every thunderclap feels like it might be the end of our lives. (36-year-old female, Slum)*

*We only want basic things – proper safety measures, drainage systems to be built, and no garbage lying around. (Female participant, BSUP)*

Participants perceived this inadequate construction as causing a sense of intrusion, discomfort, and lack of privacy within homes. This issue was mentioned to affect child-rearing practices such as breastfeeding. Participants from the BSUP colony mentioned that despite having a home to live in, the quality of the infrastructure was poor and there was little provision for maintenance of the housing.

*Two walls of our house are made of sheets, so we don’t have any windows for the air to come and go. (35-year-old female, Slum)*

*The walls of our house are also damaged due to water; if you hit the nail in the wall, it comes out (plaster of the wall) (26-year-old female, BSUP)*

#### Sub-theme 1.2. Emotional exhaustion and stress from overcrowding

Emotional exhaustion and stress stemming from overcrowding emerged in the narratives of individuals living in constrained environments, particularly in slum areas. The pervasive lack of space creates a suffocating atmosphere, where mothers articulate the challenges of maintaining privacy, especially in the vulnerable period following childbirth. This invasion of personal space breeds frustration and emotional strain, as the chaotic dynamics of extended family members moving in further complicate the living situation. Furthermore, participants frequently describe their homes as cramped, often consisting of a single room shared by an average of five family members, which amplifies feelings of overcrowding.

*My husband’s brother and his family shifted last month from our village. Since then, our house has become crowded. (28-year-old female, Slum)*

*There are four people living in the house, now, when I am pregnant, there is only one room in the house. (Female participant, Slum)*

The presence of extended family not only intensifies the physical limitations but also diminishes the sense of comfort and security, leading to a pervasive lack of privacy. For those who operate small businesses from their homes, the limited space translates into a constant source of distress, fostering a sense of helplessness amidst the chaos.

*Our family runs this tailoring business from the house itself. So, we don’t have enough space. Materials and other things are kept around. (25-year-old female, Slum)*

In contrast, while some participants from the BSUP colony also mentioned issues related to space, these concerns are less pronounced and often linked to the small size of their allotted homes rather than the overwhelming presence of extended family. Nevertheless, the overarching theme of emotional exhaustion is echoed across both groups.

### Theme 2: neighbourhood infrastructure and safety

#### Sub-theme 2.1. Fear of accidents caused by inadequate infrastructure

Nearly all participants from the slum stated that pathways were steep and narrow. Participants who were pregnant during the interview prominently mentioned that it was difficult to walk up and down the slope during pregnancy and early maternal days, as their feet would swell.

*As if you are out of breath when you climb up the slope, there is pressure in the feet too, just as the feet are swollen during pregnancy. (35-year-old female, Slum)*

*There is a problem, but in an emergency, you have to adjust yourself … now we’ve gotten into the habit too. (Female participant, Slum)*

Other associated factors mentioned were the unavailability of streetlights and safety grills along the steep slope. Participants with young children expressed their fear of potential falls. However, most participants from the slum expressed satisfaction with the daily water and electricity supply. In contrast, participants from the BSUP colony reported that these essential services were inadequate. Despite having a house to live in, participants from the BSUP colony stated that they lacked other necessities. The most commonly mentioned infrastructural needs were elevators, parks, and a reliable daily water supply. *‘Water from the tap comes only for 5–10 minutes … sometimes it doesn’t come for 4–5 days … those living on the upper floors face even more difficulty, as there is no lift and they have to use the stairs.’ (Female FGD Participant – BSUP)*

Participants highlighted additional safety concerns associated with inadequate infrastructure: *There are three floors to this building in some places the stairs are about to fall. (34-year-old female, BSUP)*

*Besides the open drains, it will also be afraid that the child will fall” (Female participant, Slum)*

#### Sub-theme 2.2. Stress related to poor sanitation, waste disposal, and environmental hazards

Many participants from the slum mentioned that there have been multiple accidents of adults and children falling into the open drainage. The issue was associated with multiple participants feeling unsafe walking around. Participants who resided in proximity to the open drainage system recounted incidents where water from the drainage overflowed into their homes during the monsoon season. This problem was commonly associated by most participants with the practice of dumping garbage into the open drainage. Other than that, garbage was mentioned to be dumped into open spaces. The main reasons stated for this practice were that garbage was not collected from doorsteps and that garbage bins were located only at the base of the slope. This problem of improper waste disposal was mentioned to be present in BSUP as well. Participants stated that the garbage collection facility was not available. The most frequently highlighted scenarios were people throwing garbage outside their house and outside the building. In contrast to these few participants, others stated that garbage collection should be available every day. Some participants also expressed the need for a daily sweeping service of the building.

*In the context of garbage being dumped in the open drainage, because of which it gets blocked during rain, and then overflows into our house sometimes. (32-year-old female, Slum)*

*The garbage collection vehicle does come, and hardly anyone gives their garbage to them. (24-year-old female, BSUP)*

#### Sub-theme 2.3. Anxiety and stress over health, accessibility, and relocation

Many participants reported a variety of health concerns, particularly anaemia, low blood pressure, and sleep disturbances as a result of caregiving obligations. Concerns about their children’s health, particularly underweight, loss of appetite, and frequent sickness, all added to their psychosocial distress.

*My daughter was good earlier, but now she has started eating clay and does not eat anything except dal rice … . she’s getting thin. (36-year-old female, Slum)*

On relocating to the BSUP colony nearly all participants expressed that place is not worth living due to unsafe and poor atmosphere of the BSUP, alternate day water supply and incidents such as suicides and thefts. Additionally, some participants expressed feeling fearful of losing their slum home to demolition or eviction. These participants also mentioned that it is better to live in the slum or relocate back to their native village than going to BSUP. Similarly, participants from BSUP prominently stated their unwillingness to live in BSUP and would rather go back to living in slum. This opinion was explained to be formed on the basis of lack of communal harmony in BSUP.

*People come and tell us that our houses will be broken, and we will also be sent to multi to live. I don’t want to go and live there (in BSUP), but if we lose this house (from slum) we will go back to our village. (21-year-old female, Slum)*

*It was better living in the slum; you at least had your people around. Go somewhere, go anywhere, leave the house of multi (BSUP). (27-year-old female, BSUP)*

### Theme 3: social cohesion, social isolation, and neighbourhood security

#### Sub-theme 3.1. Lack of communal feeling and social isolation

Most participants expressed that they do not leave the house often. Various reasons were stated for this, such as resistance from in-laws and constant fights among neighbours over day-to-day things. These were attributed to the lack of communication and social cohesion. Recurringly stated scenarios towards the same are not knowing who lives around, people coming from different places and castes, lack of opportunity for interaction. Some participants mentioned disheartening remarks about socioeconomic status from friends and relatives over housing conditions, which led to feelings of shame and inferiority. A few participants expressed that they felt a stronger sense of community feeling living around families with whom they shared similarity in terms of either native place or caste. This was also perceived by the participants to be a factor in asking for help more readily.

*If someone comes, they are like you live in a hut, what kind of place do you live in? There are no roads, the road is also useless. (22-year-old female, Slum)*

*There are different kinds of people; they keep fighting without listening to what you are saying. Like people from different states and castes, they don’t try to understand what you are trying to say. They’ll start fighting first. (26-year-old female, BSUP)*

#### Sub-theme 3.2. Fear of crime and neighbourhood insecurity

Many participants repeatedly mentioned that people engaged in activities such as drinking alcohol publicly. Additionally, the presence of thieves and stray dogs heightened anxiety about safety, as believed by the participants. A few participants expressed that reporting crimes might not lead to effective action or could even result in further harassment. Some participants associated this with unlawful activities such as selling alcohol in the BSUP colony premises. Most participants attributed their sense of fear to the presence of threatening individuals, lack of lighting in public spaces and very close proximity of homes. Additionally, other social problems were mentioned by some participants, such as suicide and fights; these were perceived by participants to cause an unsafe feeling towards residing in the BSUP colony.

*They are falling from multis (building), they hang themselves, they sell liquor. Multi is not good. (27-year-old female, BSUP)*

*If I forget to close the door, I feel panicked sometimes. People are nowadays also taking away children. So, I pay more attention to where my child is. So, we do not let the children go far away, they are always in front of our eyes. (22-year-old female, Slum)*

## Discussion

This study investigates the impact of the built environment on maternal psychosocial health among women living in slums and slum rehabilitation colonies. The findings show that overcrowding and insufficient housing conditions cause psychosocial suffering, including anxiety and weariness. Furthermore, it emphasises how poor living circumstances and safety issues add to the emotional burdens of these vulnerable populations, causing distress and a desire to leave their communities. Table 3 illustrates similarities and divergences between slums vs. rehabilitation colonies.

**Table 3.** Similarities and divergences between slums vs. rehabilitation colonies.

Common challenges in slums and BSUP colony	Challenges in slums	Challenges in the BSUP colony
**Structural vulnerability and housing safety**: Both slum and BSUP residents face issues related to inadequate housing conditions, leading to anxiety and fear. Poor construction materials and maintenance contribute to a sense of insecurity and potential structural collapse.	**Overcrowding**: Slum residents often live in cramped conditions, with multiple family members sharing small spaces, leading to emotional exhaustion and stress.	**Inadequate infrastructure**: Residents express dissatisfaction with the lack of essential services, such as a consistent water supply and proper sanitation facilities, despite having a formal housing structure.
**Neighbourhood infrastructure and safety**: Residents in both settings experience stress from overcrowding and inadequate infrastructure, such as poor sanitation and waste disposal systems. This results in health concerns and feelings of insecurity.	**Health concerns**: Slum residents report higher instances of health issues, particularly related to caregiving and children’s health, exacerbated by poor living conditions.	**Social disconnection**: The diversity of residents from different backgrounds leads to conflicts and a lack of communal ties, resulting in feelings of isolation.
**Social cohesion, social isolation and neighbourhood security**: Both groups report a lack of communal feeling and social isolation, which is exacerbated by conflicts among neighbors and a lack of opportunities for interaction.	**Fear of accidents**: The steep and narrow pathways in slums pose risks, especially for pregnant women and those with young children.	**Neighbourhood safety and security concerns**: Participants report fears related to crime and unsafe environments, including issues like public drinking and theft, which contribute to anxiety about neighbourhood safety.
	**Environmental hazards**: Open drainage systems and improper waste disposal lead to health risks and feelings of unsafety.	**Relocation anxiety**: Many residents express a preference for slum living over BSUP due to perceived safety and community ties, highlighting dissatisfaction with their current living conditions.

Respondents from both the slum and the BSUP colony identified similar elements in their housing conditions and linked them to a variety of distressing consequences, such as fear, anxiety, and a perceived lack of safety. The majority of women living in slums linked the construction of homes using inadequate materials such as metal and plastic sheets to discomfort and insecurity. These poor living circumstances, along with overcrowding owing to large family sizes and restricted space, create a sense of confinement and tension, particularly for postpartum mothers, which has been shown to influence their child-rearing techniques and relaxation.

While BSUP rehabilitation colonies were supposed to provide safer and more stable housing [6,7,11], mothers’ narratives suggested that improved housing did not always translate into increased psychosocial well-being, as seen in prior case studies from diverse BSUP contexts [8,10,17]. Poor construction quality, a lack of maintenance, and insufficient supporting infrastructure (such as lifts, water supply, and secure stairways) created new sources of stress. Several women reported a preference for returning to slums because of their greater social links, highlighting the importance of social surroundings in creating well-being. According to Debnath et al., poor design of slum rehabilitation housing and the lack of social interaction spaces contribute to discomfort and distress among residents, potentially limiting improvements in their quality of life [17].

Participants reported difficulty obtaining basic services owing to dangerous paths and inadequate sanitation. An evaluation of poverty pockets in Bhopal revealed that 40% of slum areas experienced waterlogging owing to rubbish discharge into drains, with 89% of drains being unlined [31]. Inadequate infrastructure, such as steep slope walkways and open drainages, increased the danger of falls and limited mobility, particularly for pregnant women and children. Such exterior infrastructure deficiencies exacerbated psychosocial health concerns.

The findings also highlight the importance of social cohesion, social relationships, and neighbourhood security in shaping maternal psychosocial well-being [16,32,33]. The majority of participants reported experiencing fear as a result of the presence of threatening individuals with alcohol issues, as well as incidents of suicide, theft, and fighting in both the slum and the BSUP colony. Problems with trash disposal, on the other hand, contribute to a sense of alienation based on caste, religion, and native origin. Such exclusion hindered their ability to seek assistance, as social connection is strongly relied on for immediate survival demands and health crises [34]. In line with these findings, Subbaraman, Singhi, and Parikh underline the importance of social cohesion in mitigating the adverse psychosocial effects of unfavourable living conditions. For example, Parikh et al. demonstrate how inadequate infrastructure in Indian slums disproportionately increases stress and insecurity, particularly among women, highlighting the protective role of collective action and community cohesion [14,27,35].

Generalised research on Indian urban slums [22,36–38] highlights women’s interrelated vulnerabilities as a result of gender violence and discrimination. A study conducted by Parikh [27] showed that even better basic service provision could increase hope and positive psychosocial well-being outcomes among slum dwellers. Factors such as the need to relocate to BSUP or the loss of a home in the slum increased stress. These findings are consistent with studies conducted in Mumbai slums, which discovered that criminalising the basic need for shelter and protection generates social marginalisation and affects overall wellbeing in the community [14].

While this study was originally aimed at distinguishing between mothers’ experiences in traditional slums and those in BSUP-developed rehabilitation colonies, our findings reflect a more nuanced picture. Despite the obvious structural disparities between the two dwelling types, the core psychosocial issues faced by mothers were consistent in both contexts. Inadequate waste management, social isolation, and a continual concern about child safety were issues that extended beyond the physical confines of the dwelling. This suggests that physical relocation under the BSUP programme, without concurrent improvements in social infrastructure and maintenance, may be insufficient to reduce the psychosocial burden among the urban poor. We therefore propose that maternal psychosocial well-being is influenced not only by dwelling type but also by neighbourhood infrastructure, environmental conditions, and social cohesion.

The findings provide areas for intervention to improve psychosocial health outcomes among individuals living in inadequate housing and congested situations. Policymakers should prioritise constructing safe and secure homes with suitable infrastructure, such as sanitation and waste management systems [34,39,40]. Programmes that improve neighbourhood infrastructure while promoting social cohesion and community support networks may help reduce psychosocial distress.

Furthermore, psychosocial services should be incorporated into community health activities to address recognised psychosocial stresses, particularly those associated with overcrowding, relocation, and environmental insecurity [35,41,42].

Further studies could explore ways to enable safer environments for sharing sensitive information, as mediated by trusted community facilitators. In the Indian context, larger and multi-city studies may provide broader perspectives and improve transferability of findings. Longitudinal studies on this issue may provide better comprehension of the long-term outcomes of housing upgrades on psychosocial health benefits.

### Methodological considerations

A key strength of this study lies in its qualitative design, which enabled an in-depth exploration of women’s lived experiences and perceptions of how housing and neighbourhood environments influence psychosocial health. The use of semi-structured interviews allowed participants to provide rich and detailed accounts of their daily experiences. Although the study was initially designed to include both FGDs and IDIs, challenges encountered during data collection, including participants’ reluctance to discuss sensitive issues in group settings, necessitated a greater reliance on IDIs.

Methodological and investigator triangulation were employed to enhance the credibility and trustworthiness of the findings. Data were collected through both FGDs and IDIs involving participants from diverse backgrounds. The multidisciplinary research team comprised experts in public and environmental health (VD, RRT, VY, YS), maternal and child health (UM), and qualitative research (KC, KCS, VD). The researchers held diverse academic qualifications, including PhDs (VD, RRT, KCS), MDs (UM, VY, YS), and an MPH (KC), contributing a broad range of disciplinary perspectives to the study. All researchers had formal training in research methods, while KC, KCS, and VD had specialized training in qualitative research methodologies. All the authors were employed at the Indian Council of Medical Research at the time of the study.

The FGDs and IDIs were conducted by the first author, a trained female public health researcher with prior experience in qualitative research. Other members of the research team were male researchers who contributed to study design, data interpretation, coding, and analysis. Rapport with participants was established through repeated interactions by the research team and local female community workers who facilitated community engagement and participant recruitment. Peer debriefing and member verification were undertaken throughout the analysis process to enhance the credibility and validity of the findings.

This study also has several limitations. The research was conducted within a specific urban low-income context, which may limit the transferability of findings to other settings. Furthermore, the findings are based on participants’ self-reported experiences and perceptions of psychosocial distress rather than clinical assessments of mental health outcomes. Nevertheless, these narratives provide valuable insights into how housing and neighbourhood conditions shape women’s psychosocial well-being and everyday lived experiences.

## Conclusion

The findings suggest that the built environment plays an important role in shaping maternal psychosocial well-being. Women in slum areas are especially vulnerable because they live in overcrowded, poorly built dwellings, which cause anxiety and harms their psychosocial health. The fear of accidents due to poor infrastructure and environmental stressors, such as improper trash disposal, exacerbates their psychosocial and emotional health issues. Social isolation and a lack of community cohesion in both slum and BSUP contexts cause women to feel frightened and fearful of crime, which harms their overall health. The stress of possibly moving and health issues linked with caregiving exacerbate the emotional load. The built environment has a significant impact on mothers’ psychosocial health and emotional stability. This demonstrates the importance of improving housing conditions and community support systems to create a healthy living environment. These findings provide a foundation for developing comprehensive strategies that promote resilience and psychosocial health in vulnerable populations, contributing to more inclusive and sustainable urban development.

## Supplementary Material

CPREQ_checklist.docx

SF_11.docx

## Data Availability

Data can be made available on request.
