# Supplementary material for: Women’s lived experiences of the built environment and maternal psychosocial well-being in urban slums and slum rehabilitation colonies: a qualitative study in Bhopal, India
Source: Glob Health Action. 2026 Sep 2;19(1):2721061. doi: 10.1080/16549716.2026.2721061 (PMC13539783; doi:10.1080/16549716.2026.2721061)
Supplement: Supplementary file 1 [file ZGHA_A_2721061_SM9226.docx]

### **Supplementary file**

**Understanding the Influence of Built Environment on Maternal Psychosocial Health: A Qualitative Study in Urban Poor Settings of Bhopal, India**

Topic Guide

| Name/Code | Age | Marital status | Total family member | Number of Children | Age of youngest  child | Type of house (number of rooms) | Duration of residence | Place of previous residence |
| --- | --- | --- | --- | --- | --- | --- | --- | --- |
|  |  |  |  |  |  |  |  |  |

| Sr. no | Topics of discussion | Probes |
| --- | --- | --- |
| 1. | Housing Conditions and privacy | *Location of house (top or base of the slope, floor number), construction material used, availability of toilet, sense of privacy, time for self and child care practices, if no reason, its effects* |
| 2. | Water, Sanitation and waste disposal facilities | *tap water supply, frequency, adequacy, quality, public toilet, its condition, accessibility, door to door waste collection, drainage, problems faced, incidents/examples* |
| 3. | External infrastructure | *Type of existing supporting infrastructure, condition, additional need, challenges faced during pregnancy and early maternal days* |
| 4. | Health status & Access to healthcare | *Nearby hospitals, accessibility, affordability, Health condition of self and child, health issues, perceived cause, challenges, feelings associated* |
| 5. | Interaction with community & Safety | *Frequency of interaction, type of conversation, feeling associated, reasons for affected safety,* |
| 6. | Relocation & Suggestions | *Perception on relocation, if unwilling/willing reason, perception, experience of living in BSUP or slum, other challenges and needs, preference of locality,* |

*Note: Additional probing questions asked were based on the response of the participant
